# Phosphodiesterase-4D Knockdown in the Prefrontal Cortex Alleviates Memory Deficits and Synaptic Failure in Mouse Model of Alzheimer’s Disease

**DOI:** 10.3389/fnagi.2021.722580

**Published:** 2021-09-03

**Authors:** Yongchuan Shi, Jinpeng Lv, Ling Chen, Guojun Luo, Mengjia Tao, Jianchun Pan, Xiaoxiong Hu, Jianwen Sheng, Shanjin Zhang, Min Zhou, Huizhen Fan

**Affiliations:** ^1^Department of Medicine, Jinshan Branch of the Sixth People’s Hospital of Shanghai, Shanghai Jiao Tong University, Shanghai, China; ^2^School of Pharmaceutical Engineering, Changzhou University, Changzhou, China; ^3^Key Laboratory of Clinical Cancer Pharmacology and Toxicology Research of Zhejiang Province, Department of Clinical Pharmacy, Affiliated Hangzhou First People’s Hospital, Zhejiang University School of Medicine, Hangzhou, China; ^4^School of Pharmacy, Brain Institute, Wenzhou Medical University, Wenzhou, China; ^5^Department of Gastroenterology, The People’s Hospital of Yichun City, Yi Chun University, Yichun, China

**Keywords:** PDE4D knockdown, cognitive deficits, synaptic failure, antioxidant-and antiapoptotic response, neuroprotection

## Abstract

Phosphodiesterase 4 (PDE4)-dependent cAMP signaling plays a crucial role in cognitive impairment associated with Alzheimer’s disease (AD). However, whether inhibition of PDE4 subtypes or their splice variants in the prefrontal cortex positively regulates synaptic plasticity and antioxidative stress, and reverses β-amyloid 1–42 (Aβ1–42, Aβ42)-induced cognitive impairment still need to be clarified. The present study determined whether and how PDE4D knockdown by microinjection of lenti-PDE4D-miRNA into the prefrontal cortex reversed Aβ1–42-induced cognitive impairment in behavioral, neurochemical, and molecular biology assays. The results suggested that PDE4D knockdown increased time to explore the novel object and decreased latency to leave the platform in novel object recognition and step-down passive avoidance tests. Further study suggested that PDE4D knockdown decreased the number of working memory errors in the eight-arm maze test. These effects were prevented by PKA inhibitor H89. The subsequent experiment suggested that inhibition of PDE4D in the prefrontal cortex rescued the long-term potentiation (LTP) and synaptic proteins’ expression; it also increased antioxidant response by increasing superoxide dismutase (SOD) and decreasing malondialdehyde (MDA) levels. PDE4D knockdown also increased phosphorylated cAMP response element-binding protein (pCREB), brain-derived neurotrophic factor (BNDF), and anti-apoptotic proteins’ expression, i.e., the ratio of Bcl-2/Bax, and decreased caspase-3 level in the prefrontal cortex. These findings extend the previous findings and support the hypothesis that RNA interference-mediated PDE4D knockdown in the prefrontal cortex ameliorated memory loss associated with synaptic failure in an AD mouse model by its antioxidant, anti-apoptotic, and neuroprotective properties.

## Introduction

Alzheimer’s disease (AD), the most common degenerative and irreversible brain disease causing dementia in the elderly, is characterized by progressive cognitive deterioration and memory loss (Selkoe, 2001). Clinically, pathological features of AD are extracellular senile plaques composed of fibrillar amyloid-β (Aβ) peptides and intracellular neurofibrillary tangles containing hyperphosphorylated tau, accompanied by synaptic dysfunction and neuronal death (Mangialasche et al., 2010). Although many studies are trying to find the targets for the treatment of AD, the key targets and how to control the progression of AD and related dementia are still unknown.

Phosphodiesterase-4 (PDE4), an enzyme catalyzing cAMP hydrolysis, plays a crucial role in memory consolidation and retention. Previous studies showed that PDE4 negatively regulated memory performance by decreasing hippocampal neurogenesis (Bruel-Jungerman et al., 2005; Epp et al., 2007). While inhibition of PDE4 by rolipram significantly improves hippocampal long-term potentiation (LTP) (Titus et al., 2013). Moreover, rolipram increases the levels of cAMP and phosphorylation of CREB (pCREB) (Schneider, 1984; Li, 2009), and improves learning and memory (Romano et al., 1996; Otmakhov et al., 2004) by increasing the survival and proliferation of newborn neurons in the dentate gyrus (Nakagawa et al., 2002; Fujioka et al., 2004; Sasaki et al., 2007). Indeed, PDE4 has four subtypes (PDE4A-D) that are expressed as at least 25 distinct splice variants (Houslay, 2001; Conti et al., 2003; Cheung et al., 2007; Chandrasekaran et al., 2008; Zhang, 2009). Their differential distributions in brain regions show different roles of individual PDE4 subtypes in central neuron system (CNS) disorders (Cherry and Davis, 1999; Pérez-Torres et al., 2000). For example, PDE4B is involved in depression (Millar et al., 2005) and anxiety (Zhang et al., 2008), whereas PDE4D plays an important role in memory and cognitive dysfunction (Zhang et al., 2002). Unfortunately, development of non-selective PDE4 inhibitors for therapeutic purposes has been hindered by side effects such as nausea and vomiting (Robichaud et al., 2001), which appear to be PDE4 subtype-specific (Robichaud et al., 2002). Therefore, targeting specific PDE4 isoforms, i.e., PDE4A, B, or D, could overcome this issue (Zhang et al., 2018).

The present study investigated whether inhibition of PDE4D by microinjection of lenti-PDE4D-miRNA into the prefrontal cortex of mice would rescue Aβ1–42-induced memory loss and cognitive deterioration. We found that PDE4D knockdown in the prefrontal cortex ameliorated long-term potentiation (LTP) in Aβ1–42-treated mice. The expression of Bcl-2, Bax, caspase-3, protein kinase A (PKA), phosphorylation of CREB (pCREB), and BDNF in the prefrontal cortex were also examined to decipher how PDE4D deficiency could ameliorate memory by regulation of the anti-apoptotic and neuroprotective pathway.

## Materials and Methods

### Animals

Adult male ICR mice, weighing 23–28 g, were provided by Beijing Weitong Lihua Animal Center, Chinese Academy of Sciences. The mice were raised in a temperature-controlled room with a humidity of 50–60%, natural light, circadian rhythm, and free access to food and water. The behavioral tests were carried out between 9:00 a.m. and 4:00 p.m. All procedures were carried out in a quiet room according to the “NIH Guide for the Care and Use of Laboratory Animals” (NIH Publications No. 80-23, revised 1996) and were approved by the Wenzhou Medical University Committee on Animal Care and Use and Jinshan Branch of the Sixth People’s Hospital of Shanghai, Jiao Tong University.

### Surgery

All surgery was conducted under aseptic conditions, including autoclaves of all surgery supplies before surgery and the use of a hot bead sterilizer for contaminated supplies during surgery. Mice were anesthetized with ketamine and Xylazine (100 and 10 mg/kg, i.p.) (Zhang et al., 2014). In order to relieve pain, mice were given carprofen (5 mg/kg, s.c.) before and after surgery. The fur on the top of the head was then shaved off, and the mouse was placed in a stereotaxic apparatus followed by asepsis with iodine and 70% alcohol. A 3-cm incision was made at the top of the head along the sagittal seam to expose the skull and a hole was drilled in the skull over the injection site following the coordinates (Nagai et al., 2007): Anterior-posterior (AP, from Bregma), +1.5 mm, medial-lateral (ML, from midline), ± 0.5 mm and dorsal-ventral (DV, from dura) −1.2 mm. A guide cannula (30-gauge) was implanted at the drilling site and fixed with dental tray powder. To permit diffusion after microinjection, the infusion cannula was left in place for an extra 5 min. The mice were allowed to recover for 3 days before receiving Aβ1–42 peptide (0.4 μg/μl, 1 μl/side) treatment. The lenti-PDE4D-miRNA (4DmiRNA) (5 × 10^6^ TU/μl, 1 μl/side) was microinjected 1 day after Aβ1–42 treatment. The behavioral tests were conducted 14 days after lenti-PDE4D-miRNA treatment. To determine the role of cAMP signaling in PDE4D knockdown-induced memory enhancement, PKA inhibitor H89 (2.5 μM/side) was administered for 14 days (from days 8 to 23).

### Drugs and Treatment

Aβ1–42 (rPeptide, Aβ42, United States) was dissolved in 0.9% sterile saline, at a final concentration of 0.4 μg/μl and incubated at 37°C for 4 days to obtain aggregated Aβ before micro-infusion into prefrontal cortex. H89 (Sigma-Aldrich, United States) were dissolved in artificial cerebrospinal fluid (ACSF) for prefrontal cortex microinjection from day 8 to day 22 (once a day). A total of 60 mice were randomly divided into 5 groups (12 mice in each group) for behavioral tests. The groups were normal control + vehicle, Aβ1–42 + vehicle, Aβ1–42 + 4DmiRNA, Aβ1–42 + 4DmiRNA + H89, and Aβ1–42 + H89. After behavioral tests, mice were sacrificed for LTP recording (half brain) and an immunoblot assay (the other half brain).

The primary antibodies of anti-PSD95, anti-BDNF, and anti-SYN were purchased from Abcam Biotechnology Company (Abcam, Cambridge, MA). Anti-pCREB and anti-CREB were purchased from Merck Millipore (Millipore, Billerica, MA, United States). All the secondary antibodies (anti-rabbit lgG) were purchased from MultiSciences Biotech Co., Ltd. (MultiSciences, Hangzhou, China). The Cu-Zn/MnSOD and SOD ELISA kit was purchased from Shanghai Beyotime Biological Technology Co., Ltd. (Beyotime Sciences, United Kingdom).

### Construction of Long-Form Lenti-PDE4D-miRNA

The construction of long-form PDE4D-miRNA was performed as described previously (Li et al., 2011). The sequence for the PDE4D miRNA (miRNA-mir hairpin structure) was 5-AATGGAGTCACAATCAAGTCAGTTTTGG CCACTGACTGACTGACTTGAGTGACTCCATT-3. The first 21-nts stretch is the antisense target sequence. The final sequence represents nts 1–8 and 11–21 of the sense target sequences. This miRNA sequence targets nucleotides 642–662 of the rat PDE4D4 coding sequence (GenBank accession no. AF031373), which corresponds to amino acids 214–221 within the UCR1 domain of long-form PDE4D isoforms such as PDE4D4/5; the NC sequence from Invitrogen (Carlsbad, CA, United States) was 5 -GAAATGTACTGCGCGTGGAGACGTTTTGGCCACTGACT GACGTCTCCACGCAGTACATTT-3. In both 4DmiRNA and NC sequences, the middle 19 nts (overstriking) were constant and came from the miR-155 hairpin loop. The lentiviral particles were produced by transiently transfecting HEK293T cells with a transfer plasmid containing EGFP and 4DmiRNA or NC.

### Behavioral Experiments

#### Open Field Test

The experiment was measured by an automatic activity monitoring system recording the interruption of an infrared beam (16 × 16). Each mouse was placed in the center of an autonomous activity test chamber (30 cm × 30 cm × 30 cm, with a built-in camera to record mouse activity) for accommodation for 5 min. In the test session, mouse activity was recorded within 10 min. The spontaneous activity chamber was wiped with alcohol after each test and feces were removed to avoid affecting the spontaneous activity of the next mouse (Xu et al., 2005).

#### Novel Object Recognition Test

In the acclimation session, mice were placed in a well-lighted test box (60 × 60 × 15 cm) facing the lateral wall and allowed to move freely for 5 min. The next day was a familiarization session, in which mice were placed back to the open field box, 6 cm from the side wall, and allowed to move for 5 min. There were two identical objects in this session, A1 and A2, both of which were hard weights to prevent mice from pushing and damaging them. The cumulative exploration time (A1, A2) of each object was measured, with the total time recorded as E0 and the process recorded as T0. The test session was conducted 1 h and 24 h after the familiarization session. In this session, the original object A was replaced with object B. The mice were allowed to move inside for 5 min and the cumulative exploration time (A1, B) of each object was measured. The total time was recorded as E2, and the process was recorded as T2. After each experiment, objects were wiped with 75% alcohol to avoid odor interference. The experiment was a blind test because the experimenter did not know the treatment status of the mice.

Criteria considered as exploratory behavior: (1) Mice were within 5 cm from the object; (2) mice gazed, smelt, or touched objects (head); (3) no account was taken of lying on or walking near an object. The results were expressed as discrimination index (DI), and the calculation formula was as follows: DI = B-A/E1or DI = B-A/E2 or DI = A2′-A1/E1 or DI = A2′-A1/E2 (Xu et al., 2015).

#### Step-Down Passive Avoidance Test

The test was carried out utilizing a square chamber within a wooden platform on one side of the grid floor. The grid floor of the chamber could receive an electric shock from an isolated pulse stimulator when the mice stepped down. The test included three sessions: Habituation, training, and retention. A mouse was first habituated to the apparatus for 5 min and then the training session was conducted 1 h later. In this training session, a mouse was placed on the wooden platform and was subjected to a foot shock (0.4–0.8 mA, 40 V, 0.5 s, 50 Hz, 20 sec intertribal interval) to see if it jumped off the platform with its feet fully exposed to the grid floor. The mouse was considered to have taken the task when it stayed on the platform over 60 s in the training session. Retention tests were conducted 3 h and 24 h after the training session, respectively. During the retention session, the electric shock of the grid was removed, a mouse was individually placed on the platform and the time when it first jumped off the platform was recorded as step-down latency, with an upper cutoff time of 300 s (Wang et al., 2020).

#### Eight-Arm Radial Maze (8-RAM)

The 8-RAM maze was placed 40 cm above the ground. The central area of the maze had a diameter of 30 cm and eight arms (50 cm × 12 cm, surrounded by a wall 4.5-cm high) extending at equal angles and equal lengths to all sides (Pu et al., 2007). After 1 week of acclimation, the mice were weighed and fasted for 24 h before each test. On days 8–11 of the acclimation session, food was placed in the middle and on each arm every day before animals were placed in the maze. In this session, 3–4 mice were simultaneously placed in the maze and allowed to move freely and ingest food for 10 min. Days 12–18 were the training and test session. Only four of the eight arms (arms 3, 5, 6, and 8, respectively) were fed during each exercise, which were maintained throughout the experiment. The mice were placed in the central area of the maze each day, and the doors were closed around the central area for 30 s before opening. The mice could choose to enter any arm for food intake. When the mouse entered the arm with food and received food, it was recorded as a one-time correct choice; otherwise, it was a wrong choice. Re-entering the arm with food was called a working memory error, and re-entering the arm without food was called a reference memory error. Total memory error = reference memory error + working memory error. After each experiment, the object was wiped with 75% alcohol to avoid odor interference.

#### Electrophysiological Recording

After behavioral tests, the half brain of mice was used for all field potential experiments, i.e., long-term potentiation (LTP). Slices were prepared as described previously (Misner and Sullivan, 1999). Briefly, brains were removed and coronal slices (350 mm) were cut using a vibratome (Precisionary Instruments, Greenville, NC, United States) in ice-cold solution (120 mM NaCl, 3.5 mM KCl, 0.7 mM CaCl_2_, 4 mM MgCl_2_, 1.25 mM NaH_2_PO_4_, 26 mM NaHCO_3_, 10 mM glucose) bubbled with 95% O_2_/5% CO_2_ for the electrophysiological recording. LTP of the prefrontal cortex was induced by high-frequency stimulation consisting of a single train of 100 pulses delivered at 100 Hz. This high-frequency stimulation paradigm was used to induce a short-lasting form of LTP in the control slices, so that the potentiation of LTP in other groups could be recorded (Zhang et al., 2018). Experiments and subsequent analyses were performed by investigators who were blinded to the genotype of the mice.

#### Determination of SOD Activity in the Prefrontal Cortex

The activity of superoxide dismutase (SOD, EC 1.15.1.1) was measured by monitoring its ability to inhibit the photochemical reduction of nitroblue tetrazolium (NBT). A total of 100 mM of TRIS/HCl (pH 7.8), 75 mM of NBT, 2 μM of riboflavin, 6 mM of EDTA, and 200 μL of supernatant were contained in each 1.5-mL reaction mixture. The OD value was determined in the absorbance at 560 nm (the production of blue formazan). One unit of SOD was defined as the amount which was needed to suppress the rate of NBT reduction by 50%, as previously described by Winterbourn et al. (1975). The enzyme activity was expressed as units/mg protein.

#### Determination of Malondialdehyde (MDA) Level in the Prefrontal Cortex

Malondialdehyde, an indicator of lipid peroxidation, is determined spectrophotometrically using the thiobarbituric acid analysis method according to Ohkawa et al. (1979) description. Briefly, 200 μL of supernatant was added and briefly mixed with 1 mL of 50% trichloroacetic acid in 0.1 M of HCl and 1 mL of 26 mM thiobarbituric acid. After vortex-mixing, samples were kept at 95°C for 20 min. Then, the sample was centrifuged at 960 × g for 10 min, and the supernatant was read in the absorbance at 532 nm. A calibration curve was drawn using MDA as the standard curve and the results were expressed in nmol/mg protein.

#### Western Blot Analysis

The prefrontal cortex tissues were homogenized in lysis buffer containing protease and phosphatase inhibitors and centrifuged at 12,000 rpm for 30 min at 4°C. The supernatant was mixed with loading buffer and heated to 95°C to denature the proteins. Protein concentrations were determined using the bicinchoninic acid (BCA) protein assay. The 10% sodium dodecyl sulfate polyacrylamide gel electrophoresis (SDS-PAGE) was applied to separate the protein samples (30 μg of protein/sample), and then the proteins were transferred onto a nitrocellulose filter (NC) membrane by making use of an electrophoretic transfer system (Bio-Rad, United States). Subsequently, the membranes were blocked with blocking buffer which contained 2.5% bovine serum albumin (BSA) in Tris-buffered saline and 0.1% Tween 20 (TBST) for 1.5 h at 25°C and incubated with the corresponding primary antibodies overnight at 4°C. And then the blots were incubated with peroxidase-conjugated secondary antibodies for 1 h at 25°C, followed with visualizing which used enhanced chemiluminescence.

### Statistical Analysis

All the experiments were performed in triplicate and the results were calculated by the mean ± standard error of the mean (SEM). Statistical analysis was performed according to *t*-test using GraphPad Prism 5.0 software and the value of *p* < 0.05 was considered statistically significant.

## Results

### Downregulation of PDE4D Expression by Microinjection of Lenti-PDE4D-miRNA Into the Prefrontal Cortex of Mice

**FIGURE 1:**
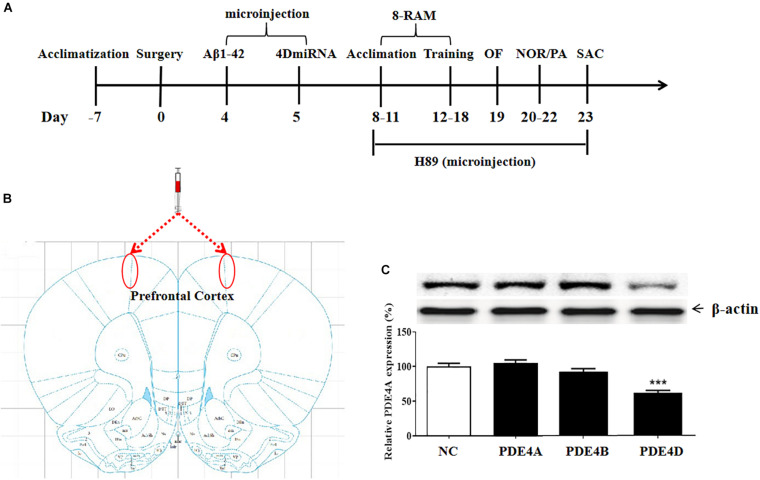
**(A)** Experimental design and the test order. Aβ 42 was microinfused into bilateral prefrontal cortices of mice on day 4 after surgery. Lentiviral vectors containing negative control sequence (NC) or 4DmiRNA were microinfused into bilateral prefrontal cortices of mice on day 5 after surgery. H89 or artificial cerebrospinal fluid were microinjected once daily starting from days 8 to 22. Behavioral experiments were performed from days 19 to 22, 30 min after H89 treatment. Animals were sacrificed for biochemical assays after behavioral tests (day 23). OF, open field test; NOR, novel object recognition test; PA, passive avoidance test; 8-RAM, eight-arm radial maze; SAC, sacrifice. **(B)** Photomicrographs of representative cannula placements in the brain region of microinjection are indicated by the red ellipse corresponding to the region. **(C)** PDE4 subtypes expression in the prefrontal cortex. PDE4D expression, but not PDE4A and PDE4B, was decreased after microinjection of lenti-PDE4D-miRNA into the prefrontal cortex for 2 weeks. Values were the means ± SEM with 12 mice in each group. ***P < 0.001 vs. vehicle-treated control group (NC).

The experimental procedure is illustrated as Figure 1A. To determine the role of long-form PDE4D variants in memory, lentiviral vectors harboring either 4DmiRs (miRNAs designed to target long-form PDE4Ds such as PDE4D4/5) or the normal control sequence were microinfused into the prefrontal cortex of mice as shown in Figure 1B. On day 23, the prefrontal cortex tissues of the mice were taken from the mouse brain for immunoblot assay. The results showed that microinjection of lenti-PDE4D-miRNA into the prefrontal cortex decreased the expression of PDE4D in the prefrontal cortex (*P* < 0.001), but did not affect PDE4A and PDE4B expression as shown in Figure 1C.

### PDE4D Knockdown Reversed Aβ1–42-Induced Memory Impairment in the Novel Object Recognition (NOR) Test

**FIGURE 2:**
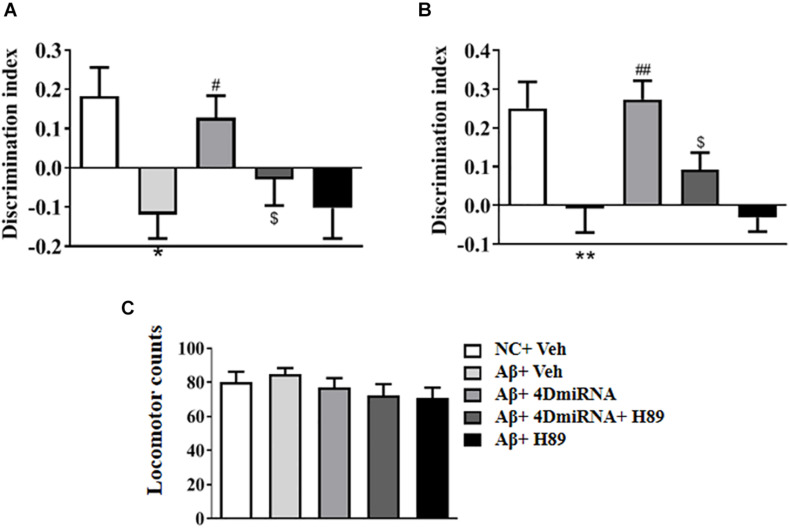
PDE4D deficiency improved cognition and memory in the novel object recognition test in Aβ1–42-treated mice. The discrimination index 3 h **(A)** and 24 h **(B)** after the training session was recorded after microinjection of lenti-PDE4D-miRNA into the prefrontal cortex for 2 weeks. In the open field test, the total distance traveled did not shown a change after PDE4D deficiency. **(C)** Values were the means ± SEM with 12 mice in each group. *P < 0.05, **P < 0.01 vs. control group (NC); ^#^*P* < 0.05, ^##^*P* < 0.01 vs. Aβ+Veh group; ^$^*P* < 0.05 vs. Aβ+4DmiRNA group.

To evaluate the therapeutic effects of PDE4D deficiency on the cognitive and memory impairment in Aβ1–42-treated mice, behavioral tests such as novel object recognition (NOR) were conducted. Mice were exposed to a pair of identical objects for 5 min, the discrimination ability was observed 3 and 24 h later, respectively. As shown in Figures 2A,B, PDE4D knockdown in the prefrontal cortex improved discrimination in the Aβ1–42-treated mice, both in 3- (*P* < 0.05; *P* < 0.05) and 24-h (*P* < 0.01; *P* < 0.01) test sessions. However, these results of PDE4D deficiency on memory enhancement were suppressed by treatment with PKA inhibitor H89 (*P* < 0.05; *P* < 0.05); while H89 treatment alone did not change the discrimination index. Importantly, the total distance animals traveled in the open field test were not found to change (Figure 2C), suggesting similar exploration and motor functioning. These findings suggest that this effect is dependent on PDE4D-PKA signaling.

### PDE4D Knockdown Reversed Aβ1–42-Induced Memory Impairment in the Step-Down Passive Avoidance (PA) Test

**FIGURE 3:**
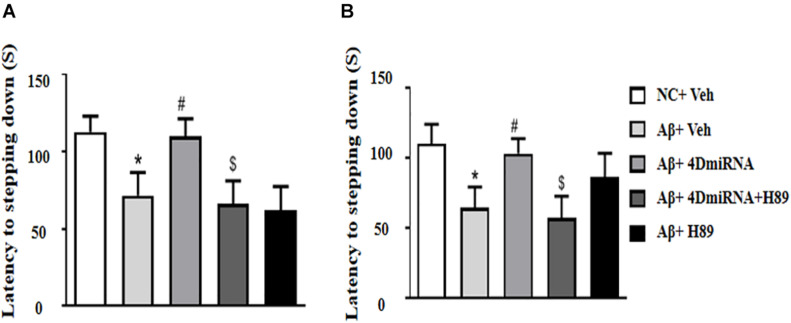
PDE4D deficiency improved memory in the step-down passive avoidance test in Aβ1–42-treated mice. The latency to jump down to the floor 3 h **(A)** and 24 h **(B)** after the training session was recorded after microinjection of lenti-PDE4D-miRNA into the prefrontal cortex. Values were the means ± SEM with 12 mice in each group. *P < 0.05 vs. control group (NC); ^#^*P* < 0.05 vs. Aβ+Veh group; ^$^*P* < 0.05 vs. Aβ+4DmiRNA group.

The memory-enhancing effect of PDE4D knockdown was determined by measurement of emotional memory performance in the PA test both in 3- and 24-h test sessions. As shown in Figures 3A,B, Aβ1–42 affected memory retention, as evidenced by the fact that the average latency to jump down from the platform was noticeably shorter, at both 3 and 24 h after training in the Aβ1–42-treated mice (*P* < 0.05; *P* < 0.05). However, the Aβ1–42-induced memory deficit was reversed in the groups treated with lenti-PDE4D-miRNA, which exhibited a greater tendency to avoid stepping onto the electrified floor as compared to the vehicle-treated Aβ1–42 group (*P* < 0.05; *P* < 0.05). This effect was prevented by pretreatment with H89 in the lenti-PDE4D-miRNA-treated Aβ1–42 group (*P* < 0.05; *P* < 0.05).

### PDE4D Knockdown Reversed Aβ1–42-Induced Memory Impairment in the Eight-Arm Maze Test

**FIGURE 4:**
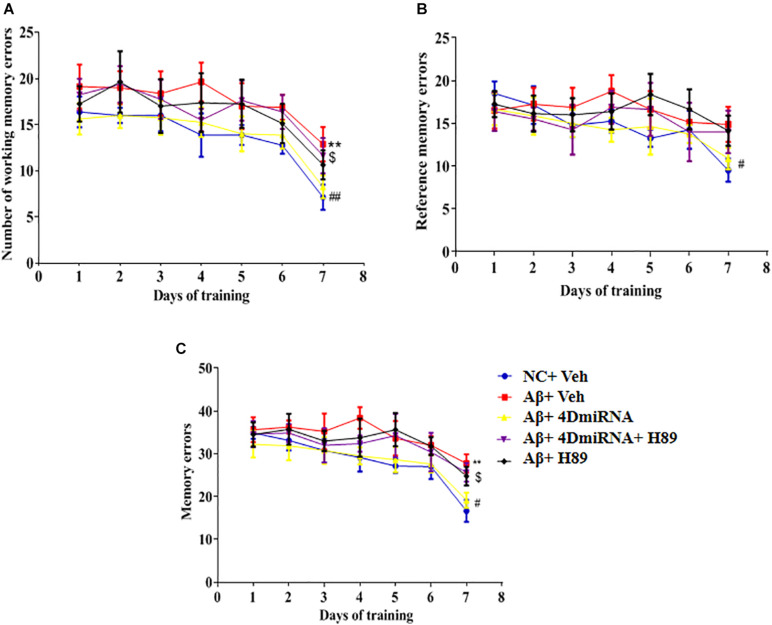
PDE4D deficiency improved memory in the eight-arm maze test in Aβ1–42-treated mice. The working memory errors, **(A)** reference memory errors, **(B)** and the total memory errors **(C)** were recorded after microinjection of lenti-PDE4D-miRNA into the prefrontal cortex. Values were the means ± SEM with 12 mice in each group. **P < 0.01 vs. control group (NC); ^#^*P* < 0.05, ^##^*P* < 0.01 vs. Aβ+Veh group; ^$^*P* < 0.05 vs. Aβ+4DmiRNA group.

As shown in Figure 4, microinjection of Aβ1–42 into the prefrontal cortex induced a significant increase in working memory errors in mice (*P* < 0.01). These Aβ1–42-induced memory defects were prevented by intra-prefrontal cortex microinjection of lenti-PDE4D-miRNA, as evidenced by a reduced number of working memory errors compared to those of the vehicle-treated Aβ1-42 group (*P* < 0.01). This finding indicates enhanced long-term memory in these Aβ1–42-treated mice. The results were consistent with the previous study (Zhang et al., 2000), which suggested that Aβ1–42-induced memory retention impairment was reversed by PDE4D knockdown in the hippocampus. Although the changes in reference memory errors were not significant between the groups throughout the training and test sessions, the total memory errors were still ameliorated when PDE4D was knocked down in the prefrontal cortex (*P* < 0.01). However, this memory enhancement of PDE4D knockdown was prevented by treatment with PKA inhibitor H89 (*P* < 0.05). While H89 used alone did not affect the memory retention of mice.

### PDE4D Knockdown Ameliorated Synaptic Plasticity and Increased Synaptic Proteins Expression in the Prefrontal Cortex of Aβ1–42-Treated Mice

**FIGURE 5:**
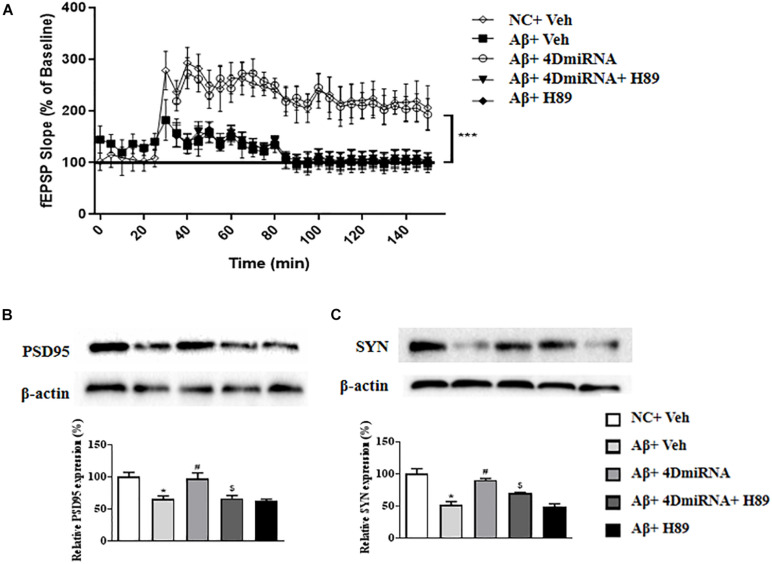
PDE4D deficiency improved LTP **(A)** and increased PSD95 and synaptophysin expression **(B,C)** in the prefrontal cortex of Aβ1–42-treated mice. Data are presented as mean ± SEM (*n* = 12); values are the means ± SEM, *P < 0.05, ***p < 0.001 vs. control group (NC); ^#^*P* < 0.05 vs. Aβ+Veh group; ^$^*P* < 0.05 vs. Aβ+4DmiRNA group.

Inhibition of PDE4 enzymes has previously been shown to enhance long-term potentiation in CA1 of the hippocampus (Barad et al., 1998; Navakkode et al., 2004). The present study showed that intra-prefrontal cortex microinjection of lenti-PDE4D-miRNA for 2–3 weeks impaired the fEPSP slope in cortex slices in the Aβ1–42-treated mice as shown in Figure 5A (*P* < 0.001). However, these results of PDE4D deficiency on LTP enhancement were prevented by treatment with H89 (*P* < 0.001). However, H89 used alone did not induce any change on LTP expression in Aβ1–42-treated mice.

As shown in Figures 5B,C, two synapses-associated proteins, e.g., synaptophysin (SYN) and PSD-95, were significantly reduced when mice were treated with Aβ1–42 (*P* < 0.05; *P* < 0.05). Microinjection of lenti-PDE4D-miRNA into the prefrontal cortex of a mouse for 2–3 weeks significantly increased the levels of synaptophysin and PSD-95 (*P* < 0.05; *P* < 0.05). However, this increased synaptic protein expression of PDE4D knockdown was prevented by treatment with H89 (*P* < 0.05; *P* < 0.05).

### PDE4D Knockdown Increased SOD Activities in the Prefrontal Cortex of Aβ1–42-Treated Mice

**FIGURE 6:**
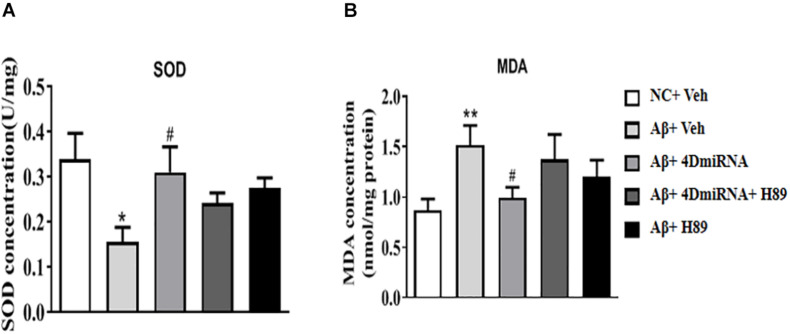
PDE4D deficiency increased SOD activity **(A)** and decreased MDA **(B)** level in Aβ1–42-treated mice. Data are presented as mean ± SEM (*n* = 12); values are the means ± SEM, *P < 0.05, **P < 0.01 vs. control group (NC); ^#^*P* < 0.05 vs. Aβ+Veh group.

Aβ1–42-treated mice were found to decrease SOD level (*P* < 0.05); while PDE4D knockdown by microinjection of lenti-PDE4D-miRNA into the prefrontal cortex rescued this decreased SOD level (*P* < 0.05; Figure 6A). These effects of PDE4D knockdown on SOD activity was partially prevented by H89, but the difference was not significant. As shown in Figure 6B, PDE4D knockdown reversed the increase in MDA level in the prefrontal cortex of Aβ1–42-treated mice (*P* < 0.05). However, this PDE4D deficiency-induced decrease in MDA level was prevented by treatment of mice with H89 (*P* < 0.05). But H89 used alone seemed not to affect either SOD or MDA expression in these mice.

### PDE4D Knockdown Increased the Levels of PKA, pCREB, BDNF, Bcl2/Bax, and Caspase-3 in the Prefrontal Cortex of Aβ1–42-Treated Mice

**FIGURE 7:**
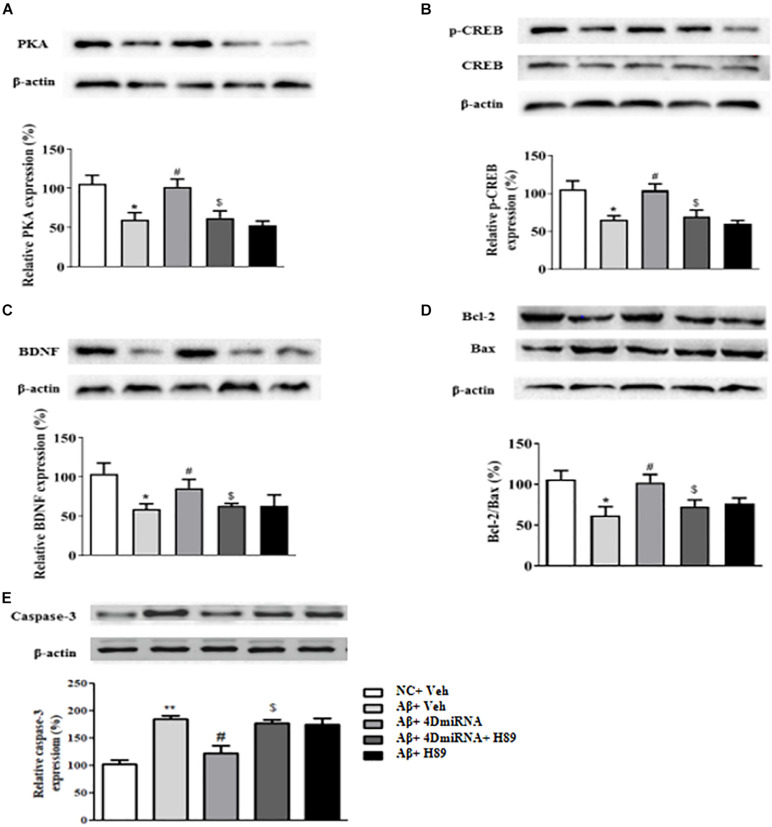
PDE4D deficiency increased expression of PKA **(A)**, p-CREB **(B)**, BDNF **(C)**, Bcl-2/Bax **(D)**, and decreased caspase-3 **(E)** level in Aβ1–42-treated mice. Results are expressed as mean ± SEM (*n* = 12). *P < 0.05, **P < 0.01 vs. control group (NC); ^#^*P* < 0.05 vs. Aβ+Veh group; ^$^*P* < 0.05 vs. Aβ+4DmiRNA group.

Given that CREB is an important downstream transcription factor of cAMP/protein kinase A (PKA) signaling and is critical for memory consolidation and synaptogenesis, we assessed PKA, CREB, pCREB, and BDNF levels in the prefrontal cortex after PDE4D deficiency in this brain region. As shown in Figure 7A, microinjection of lenti-PDE4D-miRNA into the prefrontal cortex recued the decreased expression of PKA in Aβ1–42-treated mice (*P* < 0.05). Although the total CREB levels in all groups were not significantly different, the relative pCREB expression, i.e., pCREB/CREB ratio, in the prefrontal cortex was significantly reduced in Aβ1–42-treated mice (*P* < 0.05). These effects were reversed by PDE4D deficiency in the prefrontal cortex (*P* < 0.05; Figure 7B). Moreover, the downstream BDNF expression was also decreased in Aβ1–42-treated mice (*P* < 0.05). This was also restored by microinjection of lenti-PDE4D-miRNA into the prefrontal cortex (*P* < 0.05; Figure 7C). This PDE4D deficiency-induced increased expression of PKA, pCREB, and BDNF was also prevented by H89 (*P*’s < 0.05).

Bcl-2 is an anti-apoptotic protein that inhibits activation of the apoptotic cell death pathway (Belka and Budach, 2002); while Bax is a pro-apoptotic member of the Bcl-2 protein family (Oltvai et al., 1993; Lalier et al., 2007). Cleaved caspase-3, another protein related to cell death, plays a central role in the apoptotic signaling pathway (Salvesen, 2002). As shown in Figure 7D, PDE4D deficiency reversed Aβ1–42-induced increase in Bax and decrease in Bcl-2 levels as evidenced by the increased ratio of Bcl-2/Bax in the prefrontal cortex of these mice (*P* < 0.05). As shown in Figure 7E, PDE4D deficiency reversed Aβ1–42-induced increase in caspase-3 level in the prefrontal cortex of these mice (*P* < 0.01). However, these effects of PDE4D deficiency on the ratio of Bcl-2/Bax and expression of caspase-3 were prevented by H89 (*P* < 0.05), supporting the claim that cAMP-PKA signaling is involved in PDE4D knockdown-related anti-apoptotic effects.

## Discussion

The present study suggested that PDE4D knockdown by microinjection of lenti-PDE4D-miRNA into the prefrontal cortex protects Aβ1–42-induced cognitive and memory impairment in the novel object recognition, step-down passive avoidance, and eight-arm maze tests. The results showed that PDE4D knockdown prevented neurons against oligomeric Aβ1–42 neurotoxicity, as demonstrated by preservation of LTP expression and increased synapse-associated protein expression, e.g., synaptophysin and PSD95, in the prefrontal cortex. Furthermore, PDE4D deficiency increased SOD activity and decreased MDA level. These effects are related to the increased downstream protein expression, such as PKA, pCREB/CREB, BDNF, and Bcl-2/Bax, and the decreased caspase-3 level. The protective effect of PDE4D deficiency was reversed by treatment with the PKA inhibitor H89, indicating that the beneficial effects of PDE4D deficiency are mediated through PKA activation.

Aβ accumulation is known to interfere with the cAMP-activated pathway, which plays critical roles in memory retention. Thus, the development of cAMP-enhancing strategies by inhibition of PDE4 activity, particularly PDE4 subtypes, are potential for treatment of AD and dementia. Aβ1–42 is a pivotal pathogenic factor of AD that is more susceptible to aggregation and more toxic to neurons as compared with other fragments, such as Aβ25–35 (Cheng et al., 2010). Aβ1–42 deposition in the temporal and frontal cortex is considered as an early and critical event in AD pathogenesis. The prefrontal cortex is a brain region with high expression of PDE4D (Pérez-Torres et al., 2000; Lakics et al., 2010) and importantly involved in cognition (Romanski, 2004). In the pre-experiment, Aβ1–42 was microinjected into the prefrontal cortex of the mice first, and this verified the model of AD as successful, as evidenced by the fact that Aβ1–42-treated mice showed significant memory impairment in the novel object recognition test and step-down passive avoidance test. Here we investigated PDE4D splice variants’ function using lentivirus-based RNA interference technology in the prefrontal cortex of mice. Our results suggested that PDE4D knockdown by intra-prefrontal cortex microinjection of lenti-PDE4D-miRNA decreased PDE4D expression, but did not affect PDE4A and 4B levels in the prefrontal cortex. Previous studies indicated that rolipram, a non-selective PDE4 inhibitor, prevented exogenous Aβ1–42 peptide-induced memory impairment, which may result from inhibition of long-form PDE4 (Zhang et al., 2014). We extended the study and found that Aβ1–42 peptides induced sporadic (in the novel object recognition test), spatial (in the eight-arm maze task), and emotional memory (in the step-down passive avoidance test) impairment, which can be prevented by PDE4D4/5 knockdown in the prefrontal cortex. Further study suggested that PDE4D knockdown improved cell-based learning and memory function as evidenced by ameliorating the long-term potentiation in the cortex slices of Aβ1–42-treated mice. LTP is a persistent strengthening of synapses based on recent patterns of activity, which includes one pattern of synaptic activity that produces a long-lasting increase in signal transmission between neurons (Nicoll, 2017). As memories are thought to be encoded by modification of synaptic strength, LTP is widely considered as one of the major cellular mechanisms that underlies learning and memory. The prefrontal cortex has been recognized as playing a vital role in formation of declarative memory in particular, which describes the synthesis of episodic and semantic memory (Aujla and Beninger, 2001). Our study suggested that PDE4D knockdown in the brain including the prefrontal cortex results in enhancement of episodic, spatial, and emotional memory in the novel object recognition, eight-arm maze, and step-down passive avoidance tests, which may be related to improving neuronal structure and function in the prefrontal cortex. This hypothesis was supported by the fact that PDE4D deficiency reversed Aβ1–42-induced impairment of LTP expression in the prefrontal cortex of the Aβ1–42-treated mice. The fact that PDE4D deficiency-induced amelioration on LTP was blocked by H89 suggests that the memory-enhancing effects of PDE4D inhibition may be involved in the cAMP/PKA-related downstream signaling pathway.

Increasing studies indicated that synaptic loss in the cortex is one of the crucial factors involving cognitive dysfunction (Scheff and Price, 2003; Scheff et al., 2006). The therapeutic treatments that promote synaptic plasticity may have potential benefit for patients with early or prodromal AD. Synaptophysin and PSD-95 are two synapse-associated proteins that are important for the formation of long-lasting, latent memories (Bessieres et al., 2020). Our study suggested that the levels of these two proteins were significantly decreased in Aβ1–42-treated mice, but PDE4D deficiency in the prefrontal cortex increased the expression of synaptophysin and PSD-95, indicating the role of PDE4D inhibition in amelioration of synaptic plasticity and neuronal atrophy in the progression of AD.

Recent evidence suggests that oxidative stress is involved in the mechanism of Aβ-induced neurotoxicity and AD pathogenesis (Jhoo et al., 2004). Neuronal cell exposure to Aβ was found to increase reactive oxygen species (ROS) production, which includes lipid peroxidation, protein oxidation, and the formation of hydrogen peroxide (Behl et al., 1994). In clinical investigation, researchers also found the increases of lipid peroxidation, protein carbonyl, and oxidation of mitochondrial DNA in the brain of AD patients (Lyras et al., 1997). Malondialdehyde is the most abundant individual aldehyde resulting from lipid peroxidation and can be considered as a marker of lipid peroxidation. Superoxide dismutase catalyzes the conversion of the toxic superoxide radical to less reactive hydrogen peroxide, contributing to protection from Aβ-induced neurotoxicity. In the present study, PDE4D knockdown in the prefrontal cortex increased SOD activity and decreased MDA level, suggesting that inhibition of PDE4D restored the antioxidant status of brain that may confer neuroprotection due to alleviation of oxidative damage induced by Aβ1–42. BDNF is one of the most important neuroprotective factors that plays a crucial role in repairing and maintaining neurons. The BDNF gene contains a cAMP response element (CRE) that phosphorylated CREB (pCREB) binds to enhance transcription (Yadang et al., 2020). Previous study suggested that Aβ1–42 decreased both pCREB and BDNF expression in the brain of AD mouse models (Lv et al., 2021). In our present study, PDE4D knockdown rescued Aβ1–42-induced reduction of BDNF and pCREB/CREB levels in the prefrontal cortex, which were consistent with previous studies that suggested that some PDE4 inhibitors, such as rolipram, increase pCREB/CREB ratio and BDNF levels in the frontal cortex and hippocampus of AD mice (Wimmer et al., 2020). Considering that some PDE inhibitors, such as PDE2 and PDE5 inhibitors, exhibit neuroprotective effects by regulating the balance of pro-apoptotic and anti-apoptotic processes, we also determined the role of PDE4D in regulation of imbalance of pro-apoptotic and anti-apoptotic dynamics. We examined the ratio of Bcl-2 to Bax and caspase-3 expression in the presence or absence of PDE4D inhibition in Aβ1–42-treated mice. We found that inhibition of PDE4D in the prefrontal cortex reduced apoptotic protein expression, as evidenced by an increased Bcl2/Bax ratio and decreased caspase-3 level. Further studies are ongoing in our laboratory that would decipher the detailed mechanism related to the role of PDE4D-cAMP signaling in the progression of AD.

In summary, the present study demonstrated that the memory-enhancing effects of lenti-PDE4D-miRNA may be mediated primarily via activated cAMP/CREB signaling, which may modulate several parameters of synaptic proteins, antioxidant factors, and anti-apoptotic and neuroprotective proteins. Targeting long-form PDE4D variants may be a potential target for treating memory loss associated with AD.

## Data Availability Statement

The original contributions presented in the study are included in the article/supplementary material, further inquiries can be directed to the corresponding author/s.

## Ethics Statement

The animal study was reviewed and approved by the Wenzhou Medical University Committee on Animal Care and Use and Jinshan Branch of the Sixth People’s Hospital of Shanghai, Jiao Tong University.

## Author Contributions

YS and JL: investigation, writing, and original draft. LC: writing, funding acquisition. GL: investigation and resources. MT: data curation. JP: funding acquisition. MZ: conceptualization, methodology and funding acquisition. HF: conceptualization and project administration. All authors contributed to the article and approved the submitted version.

## Conflict of Interest

The authors declare that the research was conducted in the absence of any commercial or financial relationships that could be construed as a potential conflict of interest.

## Publisher’s Note

All claims expressed in this article are solely those of the authors and do not necessarily represent those of their affiliated organizations, or those of the publisher, the editors and the reviewers. Any product that may be evaluated in this article, or claim that may be made by its manufacturer, is not guaranteed or endorsed by the publisher.
